# Mentation processes such as excessive mind wandering, rumination, and mindfulness mediate the relationship between ADHD symptoms and anxiety and depression in adults with ADHD

**DOI:** 10.1192/j.eurpsy.2023.309

**Published:** 2023-07-19

**Authors:** A. Kandeğer, S. Odabaş Ünal, M. T. Ergün

**Affiliations:** Psychiatry, Selcuk University, Konya, Türkiye

## Abstract

**Introduction:**

Excessive mind wandering is a common feature of ADHD in adulthood also referred to as mental restlessness and ceaseless mental overactivity. ADHD is a predisposing factor for many psychiatric symptoms, especially negative emotions such as depression and anxiety. Rumination is thought to be a mediator for depression in individuals with ADHD. The above-mentioned mental processes may reduce the ability of individuals with ADHD to be mindful.

**Objectives:**

This study investigates whether the mentation processes (excessive mind wandering, rumination, mindfulness) mediate the relationship between ADHD symptoms and anxiety, and depression in adults with ADHD.

**Methods:**

Medication-free 175 individuals with ADHD who were referred to the Adult Neurodevelopmental Disorders Clinic, Department of Psychiatry, Selçuk University were invited to the study. After initial diagnostic examination including The Structured Clinical Interview for DSM-5 (SCID 5), all participants completed a test battery that included a sociodemographic form, the Adult ADHD Severity Rating Scale, the Hospital Anxiety Depression Scale, the Mind Excessively Wandering Scale, the Ruminative Response Scale, and the Freiburg Mindfulness Inventory. The data of 159 patients whose ADHD diagnosis was confirmed, who did not have any mood episodes and psychotic symptoms at the time of inclusion of the study, and who filled out the forms completely, were included in the analysis. The study was approved by the Selçuk University Local Ethics Committee.

**Results:**

Participants ages ranged from 18 to 39 (mean of 22.93 ± 4.36), and 57.2% (n = 91) were women. Also, their 48.6% (n = 77) reported that they alcohol use, and 21.1% (n = 34) had substance use history. According to SCID 5 interview, participants 64.2% had comorbid psychiatric conditions. Pearson correlation analysis revealed that ADHD symptoms, rumination, excessive mind wandering, anxiety, and depression scores were significantly positively correlated with each other, but all were negatively correlated with mindfulness. Linear regression analysis showed mindfulness association with rumination, excessive mind wandering, anxiety, and depression scores, but not with ADHD symptoms. Thereupon, a conducted mediation regression analysis showed that ADHD symptoms indirectly worsened depression and anxiety through increased rumination and excessive mind wandering, and decreased mindfulness ability (Figure 1).

**Image:**

**Figure:**
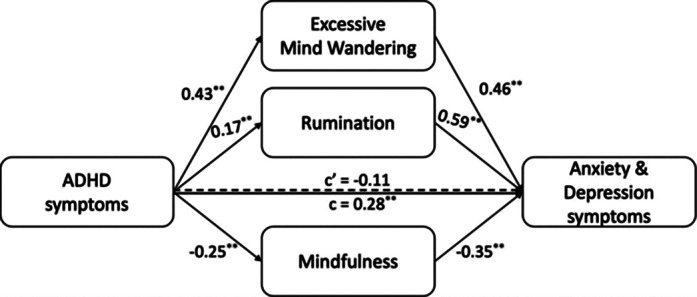

**Conclusions:**

Adults with ADHD have distressing mentation patterns which worsened their anxiety and depression. Mindfulness-based cognitive behavioral therapy modalities may help improve excessive mind wandering and rumination in ADHD. Our findings should be warranted in future studies of functional brain connectivity patterns that may serve as a mentation endophenotypes in ADHD.

**Disclosure of Interest:**

None Declared

